# Endothelial cell colony forming units derived from malignant breast diseases are resistant to tumor necrosis factor-α-induced apoptosis

**DOI:** 10.1038/srep37450

**Published:** 2016-11-24

**Authors:** Chen-Pin Chou, Shih Sheng Jiang, Huay-Ben Pan, Yi-Chen Yen, Hui-Hwa Tseng, Yu-Ting Hung, Ssu-Han Wang, Yu-Lin Chen, Ya-Wen Chen

**Affiliations:** 1 Department of Radiology, Kaohsiung Veterans General Hospital, Kaohsiung, Taiwan; 2 School of Medicine, National Yang-Ming University, Taipei, Taiwan; 3 Department of Medical Laboratory Sciences and Biotechnology, Fooyin University, Kaohsiung, Taiwan; 4 School of Medicine, National Defense Medical Center, Taipei, Taiwan; 5 National Institute of Cancer Research, National Health Research Institutes, Miaoli, Taiwan; 6 Department of Pathology, Kaohsiung Veterans General Hospital, Kaohsiung, Taiwan; 7 Program for Aging, Graduate Institute of Basic Medical Science, China Medical University, Taichung, Taiwan

## Abstract

Mobilisation of endothelial progenitor cells (EPCs) from the bone marrow is a crucial step in the formation of *de novo* blood vessels, and levels of peripheral blood EPCs have been shown to be elevated in certain malignant states. Using flow cytometry and a Hill-based colony forming unit (CFU) assay, the present study indicated that higher levels of CD34 and vascular endothelial growth factor receptor 2 (VEGFR2) double-positive EPCs, as well as increased formation of endothelial cell colony-forming units (EC-CFUs) are associated with benign and malignant breast diseases, providing possible indicators for breast disease detection. Gene expression profiles revealed a genetic difference between CD34^+^ VEGFR2^+^ EPCs and EC-CFUs. Decreased expression of tumour necrosis factor receptor 2 (TNFR2) signalling-related genes and inhibition of tumour necrosis factor (TNF)-induced signalling were demonstrated in EC-CFUs derived from patients with malignant breast disease in comparison with those from healthy controls. Interestingly, our data provided the first evidence that EC-CFUs derived from patients with malignant breast disease were resistant to TNF-α-induced apoptosis, indicating a plausible target for future therapeutic interventions.

Neoplastic survival depends on an extensive vascular network for continuous oxygen supply and also to remove toxic waste^1^. Gaining access to the host’s vascular system and maintaining a sufficient blood supply are growth-limiting steps in tumour progression. Most tumours form endothelial cell-based vessels by angiogenesis, the sprouting of new vessels from existing vessels; however, an adapted form of the embryonic process of vasculogenesis can be observed, where blood vessels arise *de novo* from endothelial precursor cells (EPCs)^2^,^3^. Recent studies have demonstrated that bone marrow-derived circulating EPCs migrate to neovascularisation sites and differentiate into endothelial cells *in situ* in the process of vasculogenesis^4^. Importantly, levels of peripheral blood EPCs have been shown to be increased in certain malignant states^5^. For instance, the amount of circulating EPCs was significantly augmented in women diagnosed with late stage invasive breast cancer^6^. After tumour resection or chemotherapy, the level of circulating EPCs becomes substantially decreased^7^,^8^. These studies demonstrated that circulating EPCs may serve as a potential tumour biomarker in breast cancer^6^,^9^.

Investigation of these EPCs has shown that a subset of pluripotent CD34^+^ stem cells has similar phenotypic features of endothelial cells. However, as EPCs and haematopoietic stem cells share many cell surface markers, including CD34, CD133, CD31, CD45, CD105, CD146, CD144, vascular endothelial growth factor receptor 2 (VEGFR2), and von Willebrand factor, the term EPC may therefore encompass a range of cells from relatively primitive haemangioblasts to more differentiated endothelial cells^10^. The EPCs were further determined to be included in the population of CD34^+^/CD133^+^ progenitor cells. In the bone marrow, early EPCs are characterised by their expression of CD34, CD133, and VEGFR2^11^. In adults, more mature EPCs are found in circulation that have lost CD133 but are still positive for VEGFR2 and CD34^12^,^13^. Based on the detection and quantification methods performed on peripheral blood samples, increased levels of circulating EPCs have been observed in malignant diseases such as lung cancer, hepatocellular carcinoma, and breast cancer^5^,^6^,^14^.

Colony-forming unit (CFU) assays have emerged as an alternative specific enumeration system for EPCs^15^. Hill *et al.* used a short-term culture assay with peripheral blood mononuclear cells grown in fibronectin-coated wells to quantify EPCs in men with a spectrum of cardiovascular risk and endothelial functions^2^. With this assay, the circulating levels of angioblast-like EPCs can be specifically quantified by their ability to form an island-like colony called a designated endothelial cell colony-forming unit (EC-CFU). Other investigators have also applied this assay to study EPCs in circulation^14^,^16^. The phenotypic characterisation of cells in EC-CFU assays has commonly been accomplished by microscopy, including visualizing the spindle-shaped appearance of cells, fluorescence staining (e.g., DiI-acetylated low-density lipoprotein (DiI-Ac-LDL) and ulex-lectin), and immunostaining, such as for CD31, a platelet-endothelial cell adhesion molecule^11^,^17^. However, additional studies have demonstrated the presence of cell surface markers for monocytes, macrophages, and lymphocytes in their culture systems, with variable evidence for endothelial growth^18^,^19^,^20^,^21^. Given the uncertainty in defining phenotypes using cellular markers or the EC-CFU assay, in the present study we aimed to determine whether the genotypic characterization of the EC-CFUs and changes in their gene expression pattern would provide insight into endothelial function and the EPC differentiation capacity between malignant breast diseases and healthy subjects. After an analysis of gene expression profiling, we observed that tumour necrosis factor (TNF)-induced signalling was down-regulated or inhibited in EC-CFUs derived from the patients with malignant breast diseases.

TNF is a pleiotropic cytokine involved in regulating diverse bodily functions, including cell growth modulation, inflammation, tumourigenesis, viral replication, and autoimmunity^22^. These functions rely on the binding of TNF to two distinct membrane receptors on target cells: tumour necrosis factor receptor 1 (TNFR1) and tumour necrosis factor receptor 2 (TNFR2). TNFR1 is ubiquitously expressed, while TNFR2 has a limited expression in certain populations of lymphocytes, endothelial cells, microglia, neuron subtypes, oligodendrocytes, cardiac myocytes, thymocytes, and human mesenchymal stem cells^23^. Typically, cells that express TNFR2 also express TNFR1, with the expression ratio varying according to cell type and functional role. Since TNFR1 typically signals cell death, while TNFR2 usually indicates cell survival, the ratio of their co-expression will shift the balance between cellular survival and apoptosis. The binding of TNF to TNFR1 triggers apoptosis through two pathways with the activation of the adaptor proteins in the TNFR1-associated death domain and the Fas-associated death domain. In contrast, TNFR2 signalling relies on TRAF2 activation and nuclear entry of the pro-survival transcription factor nuclear factor-kB.

Our study demonstrated that a higher number of EC-CFUs were derived from the peripheral blood of patients with benign and malignant breast than from healthy controls. The aim of this study was to provide a thorough, unbiased analysis of the two groups of EC-CFUs from patients with malignant breast disease and healthy controls. This was achieved using transcriptomic analysis and validated *in vitro* to distinguish the detailed molecular fingerprints of the two EC-CFUs.

## Results

### Higher numbers of CD34^+^ VEGFR2^+^ endothelial progenitor cells were observed in the peripheral blood of patients with benign and malignant breast diseases

**Figure 1:**
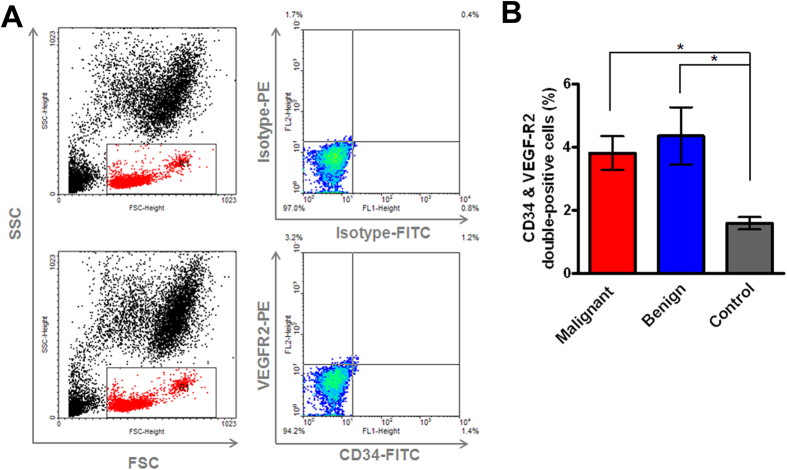
Percentages of CD34^+^ and VEGFR2^+^ cells in patients with malignant and benign breast diseases compared with that of healthy controls as determined by flow cytometery. (**A**) Forward and side scatter evaluations were used to generate the analysis gate (left panel). The upper right panel shows negative controls stained with PE- and FITC-labelled IgG isotype controls. The lower right panel contains cells stained with anti-CD34-FITC and anti-VEGFR2-PE. The percentage of the population that expressed the indicated antigens CD34^+^ and VEGFR2^+^ is shown in each quadrant. (**B**) The incidence of CD34^+^ VEGFR2^+^ cells in whole blood from patients with malignant breast diseases (n = 27), those with benign breast diseases (n = 19), and healthy controls (n = 10). Bars, SE; *p < 0.05.

Based on recent studies that have demonstrated circulating EPCs using flow cytometry^8^,^24^,^25^, the numbers of CD34^+^ VEGFR2^+^ double-positive cells were first determined in the peripheral blood of breast disease patients and healthy controls (Fig. 1A). In healthy controls, the mean percentage of circulating CD34^+^ VEGFR2^+^ EPCs was 1.59 ± 0.1912 (mean ± SE; n = 10). In benign breast disease patients, the mean proportion of double-positive EPCs was higher, with a mean value of 4.385 ± 0.9098 (mean ± SE; n = 19; *p* = 0.0385) in comparison with healthy controls. Significantly, the mean percentage of double-positive EPCs from patients with malignant breast diseases was markedly higher, with a mean value of 3.815 ± 0.5386 (mean ± SE; n = 27; *p* = 0.0185) compared to that of healthy subjects (Fig. 1B). There was no major difference in the level of CD34^+^ VEGFR2^+^ EPCs between malignant and benign breast disease patients (Fig. 1B).

### Circulating endothelial progenitor cells in breast disease patients

**Figure 2:**
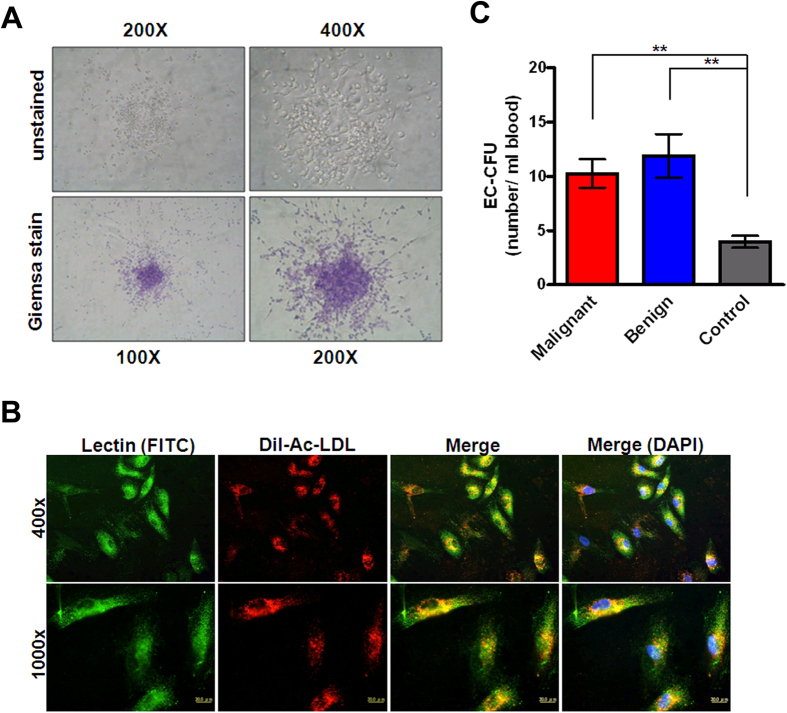
Colony-forming units of endothelial progenitor cells in the peripheral blood from patients with malignant and benign breast diseases compared with that of healthy controls. (**A**) Identification of endothelial cell colony-forming units (EC-CFUs) *in vitro* by light-field microscope. Representative EC-CFUs are shown on the seventh day of culture at 200 and 400X magnification in the upper left and right panels, respectively. Representative EC-CFUs stained with Giemsa are displayed at 100 and 200X magnification in the lower left and right panels, respectively. (**B**) Peripheral blood mononuclear cells were cultured and identified by fluorescent microscopy on the seventh day for lectin binding (*green*), 1,1′-dioctadecyl-3,3,3′, 3′-tetramethylindocarbocyanine-labelled acetylated low-density lipoprotein uptake (DiI-Ac-LDL, red), 4′,6-diamidino-2-phenylindole (DAPI, blue), and double-positive cells (yellow) are shown at 400 and 1000X magnification in the upper and lower panels, respectively. (**C**) The number of EC-CFUs per mL of whole blood from patients with malignant breast diseases (n = 30), patients with benign breast diseases (n = 20), and healthy controls (n = 11). Bars, SE; **p < 0.01.

The total mononuclear cells that had been isolated and cultured for seven days were spindle-shaped and had an endothelial-like morphology (Fig. 2A). These cells were characterised as adherent double-positive for DiI-Ac-LDL-uptake and lectin binding by confocal microscopy. Double-positive cells that appeared yellow in the overlay were identified as differentiating EPCs (Fig. 2B). The mean counts of EC-CFUs were significantly higher in patients with malignant breast diseases (10.27 ± 1.317 CFU/mL of blood; n = 30) than in healthy controls (3.955 ± 0.5539 CFU/mL of blood; n = 11; *p* < 0.01). Similarly, mean EC-CFU counts were significantly higher in patients with benign breast diseases (11.89 ± 2.012 CFU/mL of blood; n = 20; *p* < 0.01) than in healthy controls. However, we did not observe any difference in the EC-CFU counts between the groups of malignant and benign breast disease patients (Fig. 2C).

### Gene expression profiles in EC-CFUs from malignant breast cancer patients and healthy controls

**Figure 3:**
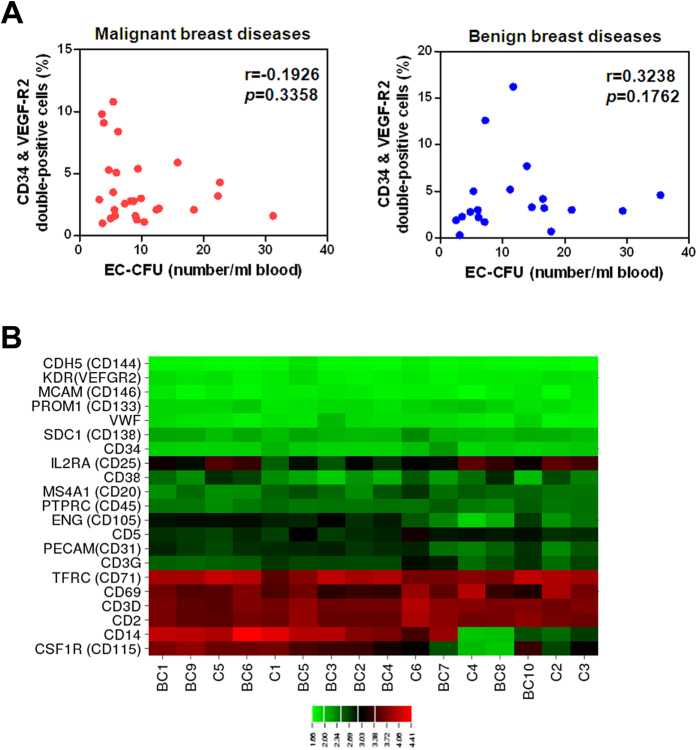
Endothelial cell colony-forming units (EC-CFUs) were genotypically different from CD34^+^ VEGFR2^+^ double-positive cells. (**A**) A scatter plot showing the correlations obtained between FACS analysis for percentages of CD34^+^ VEGFR2^+^ double-positive cells and the number of EC-CFUs from patients with malignant breast cancer (n = 27, left panel) and benign breast diseases (n = 19, right panel). (**B**) The expression of 21 cell-surface markers from 6 healthy controls and 10 malignant breast disease participants’, EPC-CFUs, ranging from 0 (green) to 4.41 (red) in log 10 transformations. Highly expressed transcripts for cell-identification markers were consistent with those of lymphocytes (CD2, CD3, CD20, CD25, CD38, CD45, CD69, and CD71).

Within the groups with malignant and benign breast disease patients, the number of EC-CFUs and the percentage of CD34^+^ VEGFR2^+^ double-positive cells failed to be correlated (Spearman r = −0.1926; *p* = 0.3358 and r = 0.3238; *p* = 0.1762, respectively), as shown in Fig. 3A. The EC-CFUs from 10 patients with malignant breast diseases (54.6 ± 3.56 years; mean age ± SE) and 6 healthy controls (46.67 ± 2.14 years; mean age ± SE) were used for transcriptome analysis using Illumina WG-6 v3.0 expression beadchips to analyse more than 48,000 transcripts, including 25,400 well characterised human transcripts and additional confirmed mRNA in the Unigene databases. To genotypically identify EC-CFUs, we examined the transcript data for cell identification markers suggested by Desai *et al.*^26^. Several highly expressed transcripts were related to markers consistent with lymphocytes, including CD2, CD3, CD20, CD25, CD69, and CD71 (Fig. 3B). The conventional endothelial marker, platelet-endothelial cell adhesion molecule (CD31), was expressed; yet other endothelial markers, such as Endoglin (ENG)/CD105, vascular endothelial cadherin (CDH5)/CD144, and von Willebrand factor, were not expressed above median values. EPC markers such as CD34, Prominin-1 (PROM1)/CD133, and kinase insert domain receptor (KDR)/VEGFR2, as well as the monocyte/macrophage marker colony stimulating factor 1 receptor (CSF1R)/CD115, were also expressed below median values (Fig. 3B).

### Differential gene expression in EC-CFUs in patients with malignant breast diseases and healthy controls

**Figure 4:**
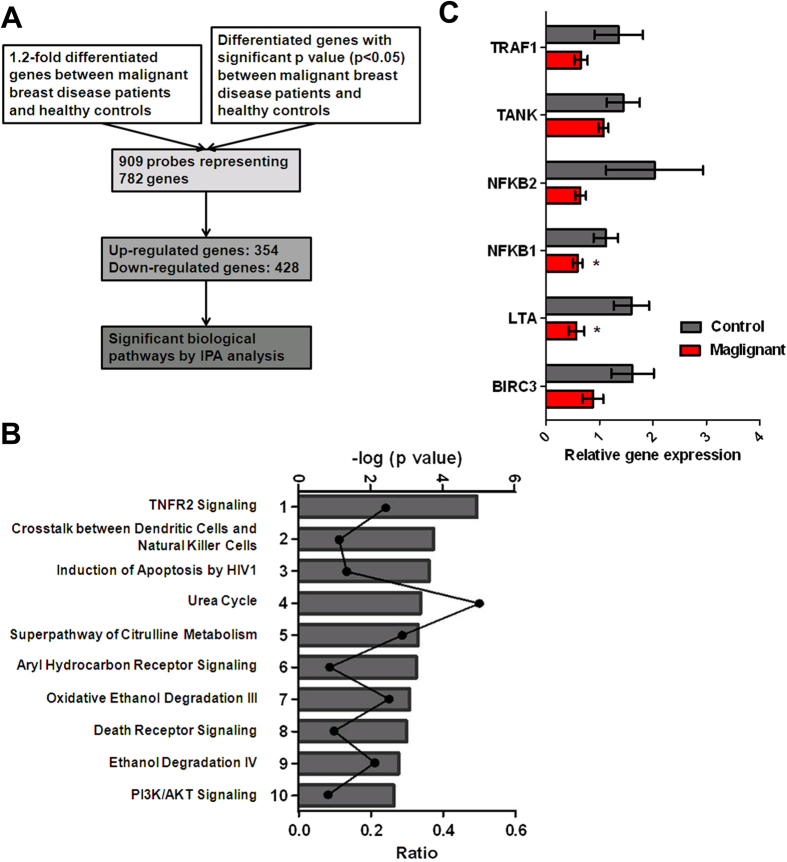
A microarray analysis of de-regulated genes among endothelial cell colony-forming units (EC-CFUs) from 6 healthy control and 10 malignant breast disease patients. (**A**) The scheme shows the data process following the microarray analysis. (**B**) Significant canonical pathways were ranked by the negative log of the calculated hypergeometric *p*-value. The curve represents the ratio of genes in the pathways. (**C**) Quantitative RT-PCR demonstrated the relative mRNA levels for BIRC3, LTA, NFKB1, NFKB2, TANK, and TRAF1. All amplifications were normalised to an endogenous β-actin control. For each gene, the relative average expression of mRNA in the EC-CFUs from five breast cancer patients was normalised to that from three healthy controls. Bar, SE *p < 0.05.

To identify the differential expression of genes between two populations of EC-CFUs from patients with malignant breast diseases and healthy controls, a total of 909 probes representing 782 genes showed at least 1.2-fold change of expression with a significant *p*-value (*p* < 0.05). From such a comparison, 354 genes were up-regulated and 428 were down-regulated in EC-CFUs from patients with malignant breast diseases when compared to those in the healthy controls (Fig. 4A). The top 10 most up- and down-regulated genes are listed in Table 1. The complete microarray data were deposited in the NCBI GEO with accession number GSE80506. In order to gain insight into the biological relevance of these genes, IPA software was used to assess the 782 differentially expressed genes. Of the 674 eligible genes, the top molecular and cellular functions of these deregulated genes were related to cellular development, cell death and survival, protein synthesis, cellular growth and proliferation, as well as cellular assembly and organization. The top canonical pathways are listed in Table 2 and Fig. 4B. Similarly, 259 genes, including 117 up-regulated and 142 down-regulated genes showed at least 1.5-fold change in expression (*p* < 0.05) in EC-CFUs from patients with malignant breast diseases when compared healthy controls (Figure S1). To validate the gene microarray results, quantitative RT-PCR (qRT-PCR) was applied to analyse gene expression in the TNFR2 pathway, which is the most significant canonical pathway after IPA using the differentially expressed genes with either 1.2 or 1.5-fold change. According to the IPA results, six highly differentiated genes including baculoviral IAP repeat containing 3 (BIRC3), lymphotoxin alpha (LTA), nuclear factor of kappa light polypeptide gene enhancer in B-cells 1 (NFKB1), TRAF family member-associated NFKB activator (TANK), nuclear factor of kappa light polypeptide gene enhancer in B-cells 2 (NFKB2), and TNF receptor-associated factor 1 (TRAF1) were chosen to analyse their expression levels between five patients with malignant breast diseases and three healthy controls. Quantitative RT-PCR revealed a lower expression of BIRC3, LTA, NFKB1, TANK, NFKB2, and TRAF1 in EC-CFUs from malignant breast disease patients in comparison with that of the healthy controls, showing consistent findings with the microarray experiments (Fig. 4C and Table 2).

### TNF-α differentially induces cell apoptosis in EC-CFUs in malignant breast disease patients and healthy controls

**Figure 5:**
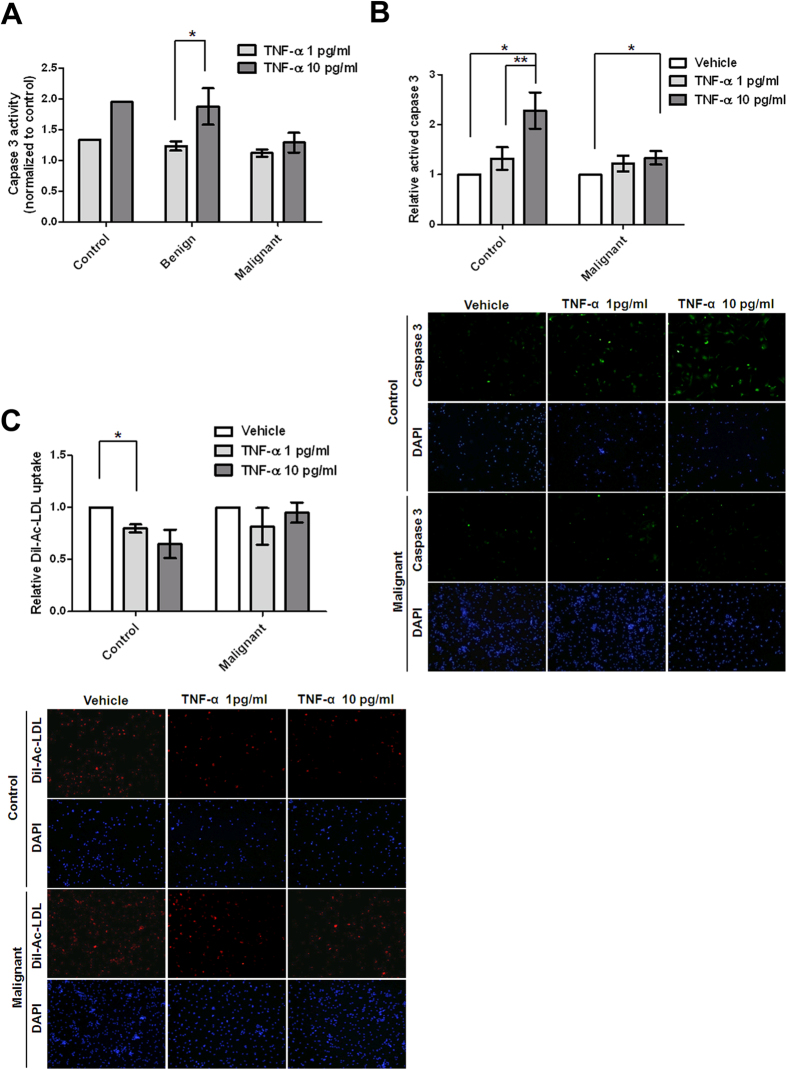
Endothelial cell colony forming units (EC-CFUs) from malignant breast disease patients were resistant to TNF-α-induced apoptosis. (**A**) The EC-CFUs were treated with a vehicle or TNF-α at 1 and 10 pg/mL for three hours. Caspase 3 activity was measured using a caspase-3 fluorogenic substrate (Ac-DEVD-AFC) by enzyme-linked immunosorbent assay. The relative activity of caspase 3 was normalised to that which received the vehicle treatment. (**B**) The active caspase 3-posive cells from 6 healthy controls and 8 malignant breast disease patients were detected using fluorescence microscopy and quantified 24 hours after the administration of TNF-α. (**C**) DiI-Ac-LDL-positive cells from 3 healthy controls and 3 malignant breast disease patients were detected using fluorescence microscopy and quantified 24 hours after the administration of TNF-α. The relative activity of caspase 3/DiI-Ac-LDL uptake in cells with TNF-α treatment was normalised to that which received the vehicle treatment. The mean of relative activity was obtained by averaging independent participants. The representative pictures of caspase 3- or DiI-Ac-LDL-positive cells were taken at 40X magnification (lower panel). Bar, SE; *p < 0.05; **p < 0.01.

Based on upstream regulator analyses suggested by IPA (Table 3), we attempted to investigate whether two populations of EC-CFUs from healthy controls and malignant breast disease patients had different responses after TNF-α treatment. To evaluate the potential influences of TNF-α on apoptosis, we analysed the activity of caspase 3 in EC-CFUs in healthy controls and also in benign and malignant breast disease patients for 3 h after the administration of TNF-α. In the EC-CFUs from healthy controls and benign breast disease patients, higher levels of caspase 3 activities were demonstrated after incubation of TNF-α at 1 and 10 pg/ml compared to those who received no TNF-α treatment. Significant levels were identified in EC-CFUs from benign breast disease patients treated with 10 pg/ml of TNF-α (n = 5, 1.875 ± 0.295, *p* = 0.0317) in comparison with those treated with 1 pg/ml (n = 5, 1.233 ± 0.0753). In contrast, we did not observe significantly increased levels of caspase 3 activity in EC-CFUs from malignant breast disease patients when they were treated with TNF-α (Fig. 5A). To confirm whether EC-CFUs of malignant breast disease patients were resistant to TNF-α-induced apoptosis and reduce the possibility of sample-specific effects, we collected different batches of whole blood only from healthy controls and patients with malignant breast disease to determine the sensitivity of TNF-α to EC-CFUs by detection of caspase 3 using fluorescent microscopy. Similarly, the means of relative caspase 3 activities demonstrated 1.32- and 2.28-fold in EC-CFUs from 6 healthy control participants treated with 1 and 10 pg/ml of TNF-α in comparison with those treated with vehicle, respectively (Fig. 5B). The data showed that TNF-α-mediated activity of caspase 3 in EC-CFUs from healthy controls was significantly enhanced in a dose-dependent manner (*p* < 0.01). In contrast, the means of relative caspase 3 activities showed 1.22- and 1.33-fold in EC-CFUs from 8 breast cancer patients treated with 1 and 10 pg/ml of TNF-α in comparison with those treated with vehicle, respectively (Fig. 5B). In comparison with low level of TNF-α, the relative activity of caspase 3 was not significantly augmented when EC-CFUs from malignant breast disease patients were treated with high level of TNF-α. Additionally, EC-CFUs were characterised as DiI-Ac-LDL-uptake by microscopy. The uptake of DiI-Ac-LDL was impaired in EC-CFUs when high levels of TNF-α were present in EC-CFUs from 3 healthy participants, whereas the uptake was not affected in EC-CFUs from 3 malignant breast disease patients (Fig. 5C). The data indicated that EC-CFUs from malignant breast disease patients were more resistant to TNF-α-induced apoptosis than those from healthy subjects.

## Discussion

Increased circulating EPC levels have been reported in various conditions associated with human vascular diseases and cancer^5^,^6^,^24^. In our study, mature EPCs from peripheral blood mononuclear cell samples were detected by flow cytometry using anti-CD34 and anti-VEGFR2. Unlike previous study^6^, most of our enrolled participants with breast diseases were asymptomatic women with abnormal screening mammogram. The levels of CD34^+^ VEGFR2^+^ EPCs from patients with early-stage breast cancer and benign proliferative breast diseases were higher than that in healthy controls (Fig. 1B). The recruited malignant breast disease patients in this study were early stage 0, I, and II. The mean percentage of double-positive EPCs from patients with stage II malignant breast diseases was higher, with a mean value of 4.323 ± 0.6551 (mean ± SE; n = 13) compared to that of stage 0-I patients with a mean of 3.343 ± 0.8459 (n = 14) (Figure S2A). Similarly, the mean number of EC-CFUs from patients with stage II malignant breast diseases was slightly higher in comparison with that of stage 0-I patients (Figure S2B). However, we did not observe a significant difference in the levels of CD34^+^ VEGFR2^+^ EPCs or EC-CFUs between stage 0-I and II malignant disease. In accordance with our findings, Dome *et al.* demonstrated higher numbers of CD34^+^ VEGFR2^+^ double-positive cells in the peripheral blood of non-small cell lung cancer patients in comparison with healthy controls and identified CD34^+^ VEGFR2^+^ double-positive cells as a progression marker^5^. Additionally, breast cancer patients with stage III and IV disease had statistically higher levels of EPCs as defined by the co-expression of CD133 and VEGFR2 than did patients with early-stage I and II disease. In late-stage patients, the levels of EPCs dropped significantly after the initiation of chemotherapy^6^. These results suggest that circulating EPCs may serve as a potential tumour biomarker in breast cancer and could represent a plausible target for future therapeutic interventions.

Unlike the use of FACS for the selection of cells with EPC markers, results from the CFU assay revealed that the selected EPCs have proliferation capacity. Indeed, it has been reported that a subset of cells derived from such CFU cell clusters were able to divide^27^. In our study, as well as in others, it was observed that the centre core of the EC-CFUs disappeared after a few days of culture, leaving behind some spindle-shaped cells, which represent cells that previously radiated out from the centre core of the EC-CFUs (Fig. 2A). In accordance with other studies, our work demonstrated significantly higher EC-CFU numbers in patients with early-stage breast cancers compared with healthy controls, illustrating that EC-CFUs may also be elevated in solid malignancies (Fig. 2C)^14^.

Ho *et al.* previously demonstrated a significant correlation between CFU scores with the FACS quantification of EPCs via labelling of early stage (CD133^+^ VEGFR2^+^) and mature (CD34^+^ VEGFR2^+^) EPC populations from the peripheral blood in hepatocellular carcinoma patients^14^. In contrast, the levels of CD34^+^ VEGFR2^+^ cells and EC-CFUs were not well correlated in our study (Fig. 3A). Using FACS or immunostaining, however, other studies have reported the presence of cell surface markers for monocytes, macrophages, and lymphocytes in their CFU culture assays, with variable evidence for endothelial growth^18^,^19^,^20^,^21^. Colonies of cells grown in fibronectin culture assays from peripheral blood mononuclear cells, believed to represent bone marrow–derived EPCs, have been associated with endothelial function and display increased levels in cancer patients^14^. However, markers of endothelial phenotype used by several groups to validate these assays—including CD31, CD105, CD144, lectin binding, and DiI-Ac-LDL uptake—are shared by many subsets of mononuclear cells^19^,^20^,^21^,^28^,^29^. Due to the uncertainty involved in phenotypic characterization using cellular markers, we considered whether genotypic characterization could serve as a more exact determinant of cellular phenotype. Our data extends these findings by using a microarray approach to genotypically determine the cellular characteristics in this assay, demonstrating that the expression of gene encoding surface markers is consistent with a T lymphocyte genotype but not with an EPC (CD34, CD133 VEGFR2) or endothelial (CD146) one. Several groups have suggested that despite the limited potential of endothelial differentiation, cells in colonies may have paracrine effects on the endothelium via the secretion of cytokines and growth factors. In this regard, our finding are consistent in part with data of Hur *et al.* and Desai *et al.*, who reported a predominance of CD3^+^ CD31^+^ cells in their colony assay^20^,^26^. They claimed that these CD3^+^ CD31^+^ cells represent a unique population of T lymphocytes that is capable of secreting angiogenic cytokines and facilitating endothelial differentiation^20^. Gene expression patterns closely matched T lymphocytes in our microarray database (Fig. 3B). Marginal expression of the transcript for CD146, a surface marker traditionally present on endothelial cells, was also noted in our microarray analysis. Flow cytometry of EC-CFUs showed a small number of CD146^+^ cells, but the majority co-expressed CD3, indicating that these cells are T lymphocytes and not endothelial cells. Lymphocyte co-expression of CD146 is representative of an activated lymphocyte phenotype with increased capacity to bind to endothelial cells and extravasate to sites of inflammation.

Previous studies have reported the capacity of anti-TNF-α treatment to improve EPC levels and to function in parallel with their clinical anti-inflammatory effect. A significant correlation was observed between the extent of clinical improvement and the level of increase in the number and function of EC-CFUs^30^,^31^. Similarly, our data showed that EC-CFUs derived from the peripheral blood of patients with malignant breast diseases were insensitive to TNF-α-induced apoptosis compared with those of healthy controls (Fig. 5), indicating that the growth of EC-CFUs was not affected by an inflammatory condition and the higher amount was observed in malignant breast diseases. Generally, TNF depends on TNFR1 for apoptosis and on TNFR2 for any function related to cell survival, although there is some degree of overlapping function depending upon the activation state of the cell and a variety of other factors^32^. In current study, there are not sufficient materials to clarify whether the level of TNFR2 signal-related gene expression in EC-CFUs is associated with the resistance of TNF-α-induced apoptosis (Figs 4C and 5A). Further investigations are needed to verify the relationships between TNFR2 signal-related gene expression and resistance of TNF-induced apoptosis in EC-CFUs.

In conclusion, here we showed that higher levels of CD34^+^ VEGFR2^+^ double-positive EPCs and endothelial colony forming cells are associated with benign proliferative and malignant breast diseases, suggesting that they may be possible indicators for disease detection. Gene expression profiles revealed the main genotypic difference between CD34^+^ VEGFR2^+^ EPCs and EC-CFUs; the differential expression profile also demonstrated a decreased expression of TNFR2 signalling-related genes in EC-CFUs from patients with malignant breast diseases. These data provide the first evidence that EC-CFUs derived from patients with malignant breast diseases were insensitive to TNF-α-induced apoptosis in comparison with those from healthy controls.

## Materials and Methods

### Patient selection

This study was HIPAA (Health Insurance Portability and Accountability Act) compliant and approved by the institutional review boards of Kaohsiung Veterans General Hospital, Taiwan (Protocol No. VGHKS13-CT8-06). A signed informed consent was obtained from every study participant before the study began. The clinical diagnosis was routinely confirmed by histopathological examination of the tissue samples. Of the 50 patients, 30 suffered from malignant breast diseases, including ductal carcinoma *in situ*, invasive ductal carcinoma, intraductal papillary carcinoma, invasive lobular carcinoma, and apocrine carcinoma. The surgical pathology revealed stage 0-II (stage 0-I, n = 16; stage II, n = 14) breast cancers (Table S1). Twenty patients, grouped in benign breast disease, had been diagnosed with proliferative benign breast diseases, flat epithelial atypia, and atypical ductal hyperplasia. The control group was comprised of 11 healthy women with matched ages. Blood samples were collected from each patient or healthy control before the treatment initiation.

### Identification of circulating EPCs from whole blood by flow cytometry

One hundred microliters of peripheral blood were stained with monoclonal antibody against VEGFR2-conjugated phycoerythrin (PE) (R&D Systems, Minneapolis MN, USA) and CD34-conjugated fluorescein isothiocyanate (FITC) (BD Biosciences, San Jose, CA, USA) at room temperature in dark conditions for 60 min. Combinations of isotype-matched immunoglobulin controls were applied as negative controls based on the species and IgG subclass of each antibody (Beckman Coulter, Brea, CA, USA). After being washed with phosphate-buffered saline (PBS) and undergoing centrifugation, the red blood cells (RBCs) were lysed using a conventional RBC lysis buffer (eBioscience, San Diego, CA, USA) and suspended in PBS. Next, the cells were divided into a two-dimensional side scatter-fluorescence histogram analysis with a fluorescence activated cell sorting (FACS) instrument (BD Biosciences). After appropriate gating with low cytoplasmic granularity and the selection of progenitor cells, all the cells that expressed CD34^+^ VEGFR2^+^ were measured. The quantity of each cell subpopulation was expressed as a percentage of the total mononuclear cells.

### Endothelial cell colony-forming unit assay

The number of EPCs was measured using the method developed in the previous study with some modifications^33^. Peripheral blood mononuclear cells were isolated using density-gradient centrifugation with Ficoll-Hypaque (GE Healthcare, Little Chalfont, UK) from 10 mL of whole blood and an ethylenediaminetetraacetic acid additive. One million mononuclear cells were suspended in a CFU-Hill liquid medium (#5900, Stemcell Technologies, Vancouver, British Columbia, Canada) in a fibronectin-coated 24-well plate (BD Biosciences). After 5–7 days of culture, the EC-CFUs were manually counted using light microscopy (Olympus BX-51, Olympus, Tokyo, Japan).

### Immunofluorescence staining

After 5–7 days of culture, fibronectin-adherent cells were washed and then incubated with 2.4 mg/ml of 1,1′-dioctadecyl-3,3,3′,3′-tetramethylindocarbocyanine (DiI)-labelled acetylated low-density lipoprotein for 4 h (DiI-Ac-LDL, L-3484, Thermo Fisher Scientific, Waltham, MA, USA). The cells were fixed for 10 min with 1% para-formaldehyde and then stained with anti-lectin antibody (ab133629, Abcam, Cambridge, UK), goat anti-rabbit fluorescein isothiocyanate (FITC, ab6717, Abcam) and 4′, 6-diamidino-2-phenylindole (DAPI, D9564, Sigma-Aldrich, St. Louis, MO, USA).

### RNA preparation

Briefly, total RNA was extracted from the cultured EC-CFUs of 10 malignant breast diseases in patients with a mean age of 54.6 ± 3.56 years and 6 healthy controls with a mean age of 46.67 ± 2.14 years using TRIzol reagent (Invitrogen, Carlsband, CA, USA). The integrity and purity of the total RNA and complementary RNA (cRNA) were analysed using an Agilent 2100 bioanalyser. Only the total RNA with a 28 S:18 S ratio of ≧1.9 was used. Complementary RNA concentrations were measured using a Nanodrop 1000 spectrophotometer (Nanodrop Technologies, Wilmington, DE, USA).

### Microarray analysis

The procedures used in this analysis have been previously described^34^. Briefly, 500 ng of the total RNA were reverse transcribed and subjected to second-strand cDNA synthesis, followed by *in vitro* transcription and complementary RNA labelling with biotin-dUTP. The labelled targets were then subjected to hybridisation with Illumina BeadChips Human HT12 according to the protocol recommended by Illumina (Illumina, San Diego, CA, USA). The chips were scanned on the Illumina BeadArray 500GX reader, and the images were processed using Illumina BeadScan software. The Genome Studio (Illumina) and Partek Genomics Suit (Partek, St. Louis, MI, USA) software packages were used for the preliminary data analysis. A model-based background correction method was employed to correct the background noise and to normalise the data before the analysis to obtain differentially expressed genes in the testing samples^35^. Microarray data are available in the Gene Expression Omnibus (GEO) database under Accession No. GSE80506. Genes with a raw signal <100 were filtered out, and the remaining genes underwent further analysis.

### Data analysis

Differentially expressed genes were selected if they demonstrated an expression fold change greater than 1.2 fold and a significant *p*-value < 0.05. Ingenuity pathway analysis (IPA, Ingenuity Systems, Redwood City, CA, USA) was used to gain an overview of the general biology that is associated with the microarray data. In the IPA, Fisher’s exact test was used to calculate a *p*-value to determine the probability that each bio-function assigned to that dataset was due to chance alone. The canonical pathway analysis tool in the IPA was used to identify signalling and metabolic pathways associated with the database. The significance of the association between the dataset and the canonical pathways was measured according to either the fold change or the *p*-value.

### Quantitative reverse transcription-polymerase chain reaction (qRT-PCR)

The qRT-PCR was performed as described previously^36^. The primer sequences that were used are listed below. BIRC3-F: 5′ TGGTTTCCAAGGTGTGAGTACTTG; BIRC3-R: 5′ AGATGAGGGTAACTGGCTTG AACT, LTA-F: 5′ ACCCCAGCAAGCAGAACTCA; LTA-R: 5′ GTTGCTCAAGGAGAAACCATCCT, NFKB1-F: 5′ GAGGTCACTCTAACGTATGCAACAG; NFKB1-R: 5′ TTGCAAGCTGCATAGCCTTCT, NFKB2-F: 5′ ACTTCTCTCCCACAGATGTG CATA, NFKB2-R: 5′ CCGCTCAATCTTCATCTTGTGA, TANK-F: 5′ CCTTTTCCCTCACTCGATTCC; TANK-R: 5′ CCACTTAGTACCACCGAGTCACTGT, TRAF1-F: 5′ AGCCTTCTACACTGCCAAGTATGG; TRAF1-R: 5′ CACGATGAAGAGCGACAGATG; β-actin-F: 5′ TGGATCAGCAAGCAGGAGTATG; and β-actin-R: 5′ GCATTGCGGTGGACGAT. All amplifications were performed in triplicate, and all values were normalised to an endogenous β-actin control. The relative expression of mRNA was also normalised with the control in each experiment.

### TNF-α treatment

Peripheral blood mononuclear cells were collected from the whole blood of participants, including healthy controls and malignant breast disease patients with stage 0-II at the time of diagnosis. After seven days of EC-CFU culture, the cells were treated with 1 or 10 pg/mL of TNF-α (R&D Systems) for three hours to induce cell apoptosis. Cell apoptosis was measured using a caspase-3 fluorometric assay kit (BF-1100, R&D Systems) or by active caspase-3 (ab13847, Abcam) immunofluorescence staining. Fluorescence levels were determined using ImageJ (National Institute of Health, Bethesda, MD, USA) and normalized against the levels of DAPI. The relative activity was determined by dividing the normalized fluorescence levels in cells with TNF-α treatment with that in cells with vehicle treatment. The mean of relative activity was obtained by averaging from all independent participants.

### Statistical analysis

The statistical analysis was performed using GraphPad software (GraphPad Software, San Diego, CA, USA). The data were expressed as means ± standard error of the mean (SE). A difference of *p* *<* 0.05 was considered statistically significant.

## Additional Information

**How to cite this article**: Chou, C.-P. *et al.* Endothelial cell colony forming units derived from malignant breast diseases are resistant to tumor necrosis factor-α-induced apoptosis. *Sci. Rep.* **6**, 37450; doi: 10.1038/srep37450 (2016).

**Publisher's note:** Springer Nature remains neutral with regard to jurisdictional claims in published maps and institutional affiliations.

## Supplementary Material

Supplementary Information

## Figures and Tables

**Table 1:** A list of the top 10 up- and down-regulated genes compiled by comparing the gene expression of the EC-CFUs from patients with malignant breast diseases with that of healthy controls.

De-regulated gene	Description	Fold change	*p* value
CD14	Cluster of differentiation 14	6.72	0.0364
CLDN23	Claudin 23	5.96	0.0339
RAB7B	Ras-related protein Rab-7b	4.81	0.0231
FOLR3	Folate receptor 3 (gamma)	4.57	0.0079
ANKRD55	Ankyrin repeat domain 55	3.87	0.0094
LRRC25	Leucine rich repeat containing 25	3.52	0.02
SPARC	Secreted protein acidic and rich in cysteine	3.24	0.0011
XYLT1	Xylosyltransferase 1	3.16	0.0035
GDF15	Growth differentiation factor 15	2.81	0.0031
HTRA4	High-temperature requirement factor A4	2.81	0.0216
IL22	Interleukin 22	0.18	0.0183
TMPRSS3	Transmembrane protease, serine 3	0.27	0.0276
EGLN3	Egl-9 family hypoxia-inducible factor 3	0.31	0.0188
TM4SF1	Transmembrane 4 L6 family member 1	0.31	0.0385
GBP6	Guanylate binding protein family, member 6	0.31	0.0308
SDK2	Sidekick cell adhesion molecule 2	0.31	0.036
UBD	Ubiquitin D	0.31	0.0377
IL26	Interleukin 26	0.33	0.0286
IQCG	IQ motif containing G	0.34	0.0455
FEZ1	Fasciculation and elongation protein zeta 1	0.35	0.014

**Table 2:** Differentially expressed genes in the most relevant biologic pathways identified by IPA.

Biological pathways	Underexpressed genes	Fold change	Overexpressed genes	Fold change
TNFR2 Signaling	BIRC3	0.63	NAIP	1.41
	LTA	0.53		
	NFKB1	0.71		
	NFKB2	0.57		
	TANK	0.73		
	TRAF1	0.58		
Crosstalk between Dendritic Cells and Natural Killer Cells	CD226	0.73	CAMK2B	1.65
	FAS	0.57		
	HLA-A	0.7		
	IL15RA	0.36		
	IL2R	0.68		
	IL2RG	0.72		
	LTA	0.53		
	NFKB1	0.71		
	NFKB2	0.57		
Induction of Apoptosis by HIV1	BIRC3	0.63	NAIP	1.41
	FAS	0.57	TNFRSF1A	1.34
	NFKB1	0.71		
	NFKB2	0.57		
	SLC25A4	0.62		
	TRAF1	0.58		
Urea Cycle			ARG1	1.75
			ASL	1.32
			ASS1	1.29
Superpathway of Citrulline Metabolism	ALDH18A1	0.74	ARG1	1.75
			ASL	1.32
			ASS1	1.29

**Table 3:** A list of the significant upstream regulators.

Upstream Regulator	Molecule Type	Predicted Activation State	Activation z-score	p-value of overlap
STAT5A	transcription regulator		−0.610	4.54E-06
NFkB (complex)	complex	Inhibited	−3.165	1.23E-05
MAP3K14	kinase	Inhibited	−2.800	4.59E-05
PSMB9	peptidase			5.21E-05
TNF	cytokine	Inhibited	−2.361	5.53E-05
