# Supplementary material for: Endothelial cell colony forming units derived from malignant breast diseases are resistant to tumor necrosis factor-α-induced apoptosis
Source: Sci Rep. 2016 Nov 24;6:37450. doi: 10.1038/srep37450 (PMC5121583; doi:10.1038/srep37450)
Supplement: Supplementary Information [file srep37450-s1.pdf]

Endothelial cell colony forming units derived from malignant breast diseases are resistant to tumor necrosis factor-α-induced apoptosis

Chen-Pin Chou , Shih Sheng Jiang, Huay-Ben Pan, Yi-Chen Yen, Hui-Hwa Tseng, Yu-Ting Hung, Ssu-Han Wang, Yu-Lin Chen, Ya-Wen Chen

Supplementary data

Table S1. Clinicopathologic parameters of 50 women with breast diseases

Clinical parameter	No. (%)
**Age**	
≧50	17 (34)
＜50	23 (66)
**Pathology Type**	
Malignancy	30 (60)
Benign	20 (40)
**TNM cancer stage (30 malignancy)**	
0	8 (27)
I	8 (27)
II	14 (46)
**Histology (30 malignancy)**	
Invasive carcinoma	22 (73)
Non-invasive carcinoma	8 (27)

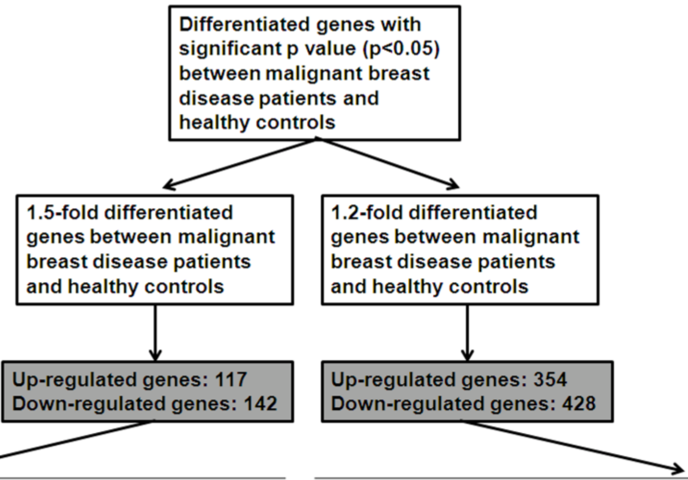

Name of top Canonical pathways (259 geens)	P value	overlap
TNFR2 signaling	2.24E-04	14.3% (4/28)
Altered T cell and B cell signaling in rheumatoid arthritis	2.50E-04	7.4% (6/81)
Induction of apoptosis by HIV1	4.45E-04	8.5% (5/59)
Histamine degradation	5.19E-04	20% (3/15)
Oxidative ethanol degradation III	7.64E-04	17.6% (3/17)

Name of top Canonical pathways (782 genes)	P value	overlap
TNFR2 signaling	1.10E-05	24.1% (7/29)
Crosstalk between dendritic cells and natural killer cells	1.73E-04	11.2% (10/89)
Induction of apoptosis by HIV1	2.35E-04	13.3% (8/60)
Urea cycle	3.95E-04	50% (3/6)
Superpathway of citrulline metabolism	4.65E-04	28.6% (4/14)

Figure S1. Ingenuity pathway analysis (IPA) of de-regulated genes with high and low fold change among endothelial cell colony-forming units (EC-CFUs) from healthy controls and malignant breast disease patients.

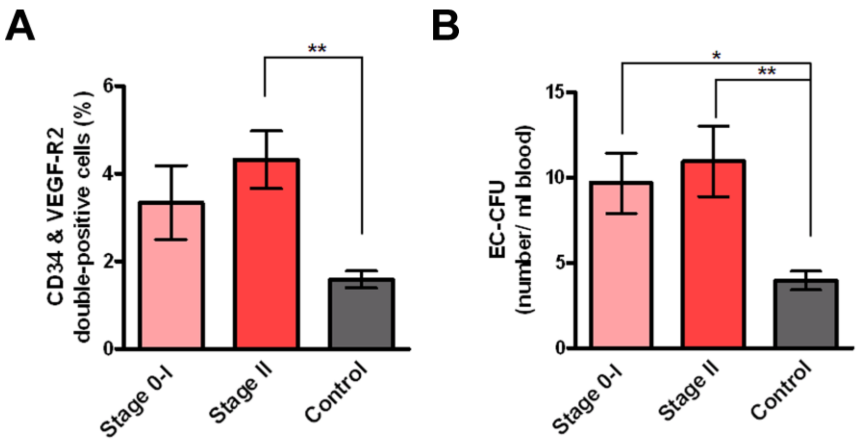

Figure S2. Comparison of levels of $CD34^{+}VEGFR2^{+}$ cells and endothelial cell colony-forming units in malignant breast disease patients with stage 0-I and II. (A) The incidence of $CD34^{+}VEGFR2^{+}$ cells in whole blood from patients with stage 0-I (n=14), stage II (n=13) malignant breast diseases, and healthy controls (n=10). (B) The number of EC-CFUs per ml of whole blood from patients with stage 0-I (n=16), stage II (n=14) malignant breast diseases, and healthy controls (n=11). Bars, SE; *p<0.05; **p<0.01.
